# The Role of Inhibitory Control, Attention and Vocabulary in Physical Aggression Trajectories From Infancy to Toddlerhood

**DOI:** 10.3389/fpsyg.2020.01079

**Published:** 2020-05-26

**Authors:** Dide S. van Adrichem, Stephan C. J. Huijbregts, Kristiaan B. van der Heijden, Stephanie H. M. van Goozen, Hanna Swaab

**Affiliations:** ^1^Clinical Neurodevelopmental Sciences, Leiden University, Leiden, Netherlands; ^2^Leiden Institute for Brain and Cognition, Leiden University, Leiden, Netherlands; ^3^School of Psychology, Cardiff University, Cardiff, United Kingdom

**Keywords:** physical aggression, inhibitory control, attention, vocabulary, infancy, toddlerhood

## Abstract

Physical aggression has its origin very early in development, but no studies to date have examined physical aggression trajectories starting before the age of 1.5 years. This study examined whether cognition plays a role in the development of physical aggression from infancy onward. In a sample of 182 mother-child dyads (94 boys; 88 girls), child physical aggression was assessed by maternal report using the Physical Aggression Scale for Early Childhood at 12, 20, and 30 months. Children performed cognitive tasks measuring inhibitory control and attention, and mothers rated children’s vocabulary at 12 and 30 months. Results showed that differential development of physical aggression already starts at 12 months of age: low-stable, low-increasing, moderate-decreasing and high-stable trajectory groups were identified. Inhibitory control, attention and vocabulary at 12 months and development of these abilities from 12 to 30 months were selectively related to the likelihood of following the low-increasing and moderate-decreasing trajectories compared to the low-stable physical aggression trajectory. This study is the first to show that specific aspects of cognition and cognitive development are related to differential physical aggression development from infancy onward.

## Introduction

Studies examining children during preschool and school age indicate that delays in cognitive development are related to higher levels of physical aggression and externalizing behavior problems (Schoemaker et al., 2013; Girard et al., 2014). Although physical aggression emerges in typical development during the first year of life, research examining the relation between cognition and physical aggression during infancy and toddlerhood is scarce. High and persistent levels of physical aggression during early childhood are predictive of negative outcomes later in life, such as ongoing externalizing behavior problems, delinquency, problems with peers, lower academic achievement, and internalizing problems (Mesman et al., 2001; Broidy et al., 2003; Masten et al., 2005; Campbell et al., 2006). In order to identify opportunities for early intervention programs to prevent such negative developmental outcomes, the current study examined whether cognitive factors in infancy and toddlerhood were related to distinct physical aggression trajectories between the ages of 12 and 30 months.

The use of physical aggression is considered normal and adaptive during the first years of life (Tremblay and Nagin, 2005). During the first half year of life, infants express frustration and anger through vocalizing and facial expressions (Hay, 2017). The early manifestations of physical aggression are apparent before 12 months of age, as soon as children acquire the motor skills to use force (Hay et al., 2010). The first expressions of aggressive behavior involve behaviors such as biting, hitting, pulling, and tugging toys: these behaviors are generally considered to be reactive in nature and to result from frustration (Hay, 2017), while harmful intent is very unlikely to play a role in infant aggression (Gendreau and Archer, 2005). From infancy onward, the level of physical aggression increases until it peaks around 2 years of age (Alink et al., 2006; Nærde et al., 2014). During the second year of life, aggressive behavior begins to include more instrumental components with the intention to achieve a goal, such as acquiring or defending toys (Hay et al., 2011). Due to, among others, the development of verbal abilities and inhibitory control during toddlerhood, children learn to control their emotions and behavior (Garon et al., 2008). As a result, the level of physical aggressive behavior starts declining from around 2 years of age (Côté et al., 2006; Nærde et al., 2014).

However, not all children follow this most common pattern of physical aggression development. Studies of distinct trajectories of physical aggression and externalizing behavior problems have focused mainly on development during preschool age, school age, and adolescence (Broidy et al., 2003; Bongers et al., 2004; Jennings and Reingle, 2012; Olson et al., 2017). Studies identifying distinctive physical aggression trajectories starting at age two until school age consistently find that a majority of children indeed show a decrease in aggressive behavior, but also indicate the existence of a high persistent developmental pattern during early childhood (Campbell et al., 2006; Côté et al., 2006). Several studies examining physical aggression trajectories starting in toddlerhood (at 1.5 years old) also found a low-stable trajectory, in addition to a moderate and increasing or moderate and stable physical aggression trajectory and a high increasing or stable pattern until 3 or 3.5 years of age (Tremblay et al., 2004; Huijbregts et al., 2008; Wildeboer et al., 2015; Mazza et al., 2017). A study focusing on the trajectories of individual aggressive behaviors between age 8 and 24 months identified two classes: children with a relatively high prevalence and children with a relatively low prevalence of specific aggressive behavior forms (Lorber et al., 2018). Although aggressive acts are already present during infancy, no studies to date have examined specific physical aggression trajectories starting before the age of 1.5 years. It is unknown whether a high level of physical aggressive behavior during toddlerhood reflects the continuation of a behavioral pattern starting during the first year of life. In addition, because of the importance of intervening at the earliest possible developmental stages (Doyle et al., 2009), this study examined physical aggression trajectories from age 12 months up to 30 months.

Self-regulation consists of physiological, cognitive, and emotional processes that play a central role in behavioral functioning (Calkins and Keane, 2009). Deficits in these regulatory systems have been found to negatively influence children’s ability to deal with challenging situations. Although it should be noted that the regulatory processes, including cognitive processes and emotion regulation, mutually influence and support each other (Blair and Diamond, 2008), studies indicated that the regulatory systems can be differentiated during early childhood (Blankson et al., 2012). Our study focused on cognitive functions, which have been proposed as mechanisms explaining brain-behavior relations: dysfunctions in neurobiology are reflected in problems in cognitive functioning, which lead to behavior problems (Bishop and Snowling, 2004; Beauchamp and Anderson, 2010).

Several cognitive functions have been related to aggressive and externalizing behavior problems during toddlerhood, preschool, and school age (e.g., Bellanti and Bierman, 2000; Estrem, 2005; Towe-Goodman et al., 2011; Spann and Gagne, 2016). Although both the cognitive processes and aggression start to develop from infancy onward (Garon et al., 2008; Hay et al., 2010), less is known regarding the relation between physical aggression and cognitive development before preschool. Therefore, we examined the relations between the development of physical aggression and three cognitive functions during infancy and toddlerhood: inhibitory control, vocabulary, and attention.

Inhibitory control is the capacity to suppress a dominant or prepotent response (Garon et al., 2008). The development of inhibitory control starts in the first year of life (Kochanska et al., 1998; Garon et al., 2008). Kochanska et al. (1998) showed that 8-to-10-month-old children are able to suppress the tendency to touch an attractive object when requested by a parent. Subsequently, inhibitory control rapidly develops during toddlerhood and the preschool period: children become better at delaying their response for a (larger) reward (Kochanska et al., 2000; Carlson, 2005), and at following arbitrary rules in conflict-tasks, in which (motor or verbal) responses have to be inhibited in certain situations, or in which an automatic response has to be inhibited to provide a non-dominant response (Carlson, 2005).

Inhibitory control is one of the core executive functions, which are the cognitive processes involved in regulating behavior in an effective and goal-oriented way (Garon et al., 2008). These cognitive processes are important to solve novel problems and adapt to new (social) situations. In line with this, impairments in inhibitory control have been related to aggressive behavior in childhood (Ellis et al., 2009). Studies also consistently show the relation between inhibitory control and externalizing behavior problems or physical aggression during preschool, using both neurocognitive tasks and questionnaires to examine inhibitory control (Schoemaker et al., 2013; Suurland et al., 2016; Olson et al., 2017). One of the few studies examining the relation between effortful control (an umbrella term for different abilities, including inhibitory control, required for goal-directed and socially adaptive behavior) and externalizing behavior before preschool showed that better effortful control at 22 months was related to less expressed anger at 33 months (Kochanska et al., 2000).

Acquiring language skills is another very important aspect of cognitive development during the first years of life. As early as 5 months old, infants are able to recognize their own name (Delle Luche et al., 2017) and start associating sounds and words with objects (Gogate and Hollich, 2010). During the second half of the first year, infants start vocalizing and babbling (Lee et al., 2017), followed by using words around their first birthday (Rose et al., 2009). From toddlerhood, children undergo a rapid development in the amount of words they can produce (Dale and Goodman, 2005).

It has been suggested that the development of language enables children to communicate with their social environment about their desires and needs, resulting in less frustration and fewer behavior problems (Keenan and Shaw, 1997). In line with this hypothesis, both language comprehension and production have been negatively related to externalizing behavior problems and physical aggression during preschool and school age (Estrem, 2005; Menting et al., 2011; Petersen et al., 2013). Although some studies found evidence for a language ability-aggression relation at around 18 months (Dionne et al., 2003; Girard et al., 2014), research into this association during infancy and early toddlerhood is limited. Reason for this might be that the assessment of language development this early (i.e., during infancy), which focuses on babbling, vocalizing, and comprehension and production of first words (Lipkind et al., 2019), is very complex in itself, thereby leaving a much smaller number of studies that link these abilities to behavior problems.

The abilities to orient to a stimulus and to focus attention are present in rudimentary form at birth (Colombo, 2001). During the first months of life, attention is largely determined by novelty of objects and events. Toward the end of the first year, novelty becomes less important in governing attention, which enables children to control their attention more voluntarily (Colombo, 2002). As a result, infants become able to shift their attention between objects (Rothbart and Posner, 2001) and to focus and sustain their attention for longer periods of time (Colombo, 2001; Ruff and Capozzoli, 2003).

In addition to inhibitory control and vocabulary, attention appears to be another important construct involved in the development of aggressive behavior. Sustained attention has been proposed as a necessary skill to be able to recognize and appropriately respond to social cues in interaction with others (Murphy et al., 2007). Children with problems in maintaining attention will probably miss social signals, resulting in less social competence and higher levels of behavior problems. In line with this, studies with children at preschool and school age have confirmed the relation between problems with focusing and sustaining attention and externalizing behavior problems (Bellanti and Bierman, 2000; Towe-Goodman et al., 2011). In addition, inattention at age two has been related to a chronic trajectory of externalizing behavior problems during toddlerhood and preschool (Hill et al., 2006). However, we do not yet know whether infant attention and the development of attention up to toddlerhood is related to physical aggression trajectories.

The current study extends the existing literature on the development of physical aggression by examining the predictive role of infant cognition. Our study focused on physical aggression specifically, because this is the first type of aggression that can be observed during early development. Based on the existing literature, it was first hypothesized that three trajectories would provide the best-fitting model for physical aggression development between age 12 and 30 months: a low-stable trajectory, a moderate-increasing trajectory, and a high-increasing or high-stable trajectory. Second, lower inhibitory control, attention and vocabulary at 12 months were expected to result in higher chances of following the moderate-increasing or high-increasing/high-stable trajectories compared to the low-stable trajectory. Third, it was expected that higher chances of following the moderate-increasing or high-increasing/high-stable trajectories of physical aggression would be related to a less optimal development of cognition between age 12 and 30 months.

## Materials and Methods

### Participants

Participants were part of the Mother–Infant Neurodevelopment Study in Leiden (MINDS-Leiden, Netherlands; Smaling et al., 2015; Suurland et al., 2017), a longitudinal study following mother-child dyads from pregnancy until the child is almost 4 years old to examine the neurobiological and neurocognitive predictors of early behavior problems. Dutch-speaking women who were between 17 and 25 years old and expecting their first child were eligible to participate. Recruitment of pregnant woman took place via hospitals, midwifery clinics, prenatal classes, and pregnancy fairs. For this study, data of the third (12 months post-partum), fourth (20 months post-partum) and fifth data waves (30 months post-partum) was used.

Of the 210 recruited participants, 13% (*n* = 28) dropped out before the assessment at 12 months. Drop-out was unrelated to maternal age and ethnicity, but mothers who dropped out tended to be more often single, χ^2^(1) = 2.38, *p* = 0.06, and tended to have a lower average family income, *t*(204) = −1.70, *p* = 0.09, compared to mothers who completed the study. The final sample consisted of 182 mother-child dyads (94 boys, 51.6%). Average age of mothers at the first assessment (during pregnancy) was 22.9 years (SD = 2.1). Average age of the children was 12.53 months (SD = 0.61) at the third wave, 20.43 months (SD = 0.73) at the fourth wave and 30.61 months (SD = 1.02) at the fifth wave. The majority of the mothers were Caucasian (87.9%), the remaining mothers were Surinam or Antillean (3.0%), and from other origin (9.1%). With regard to marital status, 11.5% of the mothers were single. Mean family income was 2596 Euros per month (SD = 1137).

### Procedures and Instruments

The study was approved by the ethics committee of the Department of Education and Child Studies at the Faculty of Social and Behavioral Sciences, Leiden University (ECPW-2011/025), and by the Medical Research Ethics Committee at Leiden University Medical Centre (NL39303.058.12). All participating women provided informed consent.

#### Physical Aggression at 12, 20, and 30 Months

The Physical Aggression Scale for Early Childhood (PASEC) was completed by the mother to examine physical aggression (Alink et al., 2006). The PASEC consists of 11 items (for example, *starts fights* and *hits*), based on the behavioral questionnaire by Tremblay et al. (1999) and the Childhood Behavior Checklist 1½-5 (Achenbach and Rescorla, 2000), and examines the presence of physical aggression during the past 2 months using a 3-point Likert scale (0 = not true, 1 = somewhat or sometimes true, 2 = very true or often true). A total score for physical aggression was calculated by summing the item scores (possible range: 0–22). Inter-rater reliability, convergent validity and 1-year stability were substantial in a Dutch sample (Alink et al., 2006; Mesman et al., 2008). Cronbach’s α for the present sample was 0.75 at 12 months, 0.74 at 20 months, and 0.73 at 30 months. Data regarding physical aggression was missing for 10 children at 12 months, 5 children at 20 months, and 32 children at 30 months, because mothers did not participate in the corresponding data wave.

#### Inhibitory Control at 12 Months

Inhibitory control was measured at 12 months using an adapted version of the Don’t paradigm (Kochanska et al., 1998). During the 2-min task, the mother was asked to prohibit her child from touching an attractive toy (with colors, sounds, and lights) using words and sounds she used in daily settings (for example, “no-no” and “don’t do that”). The behavior of the infant was filmed and was coded afterward following the coding manual of Kochanska and Aksan (2005), coding 24 segments of 5 s each using five mutually exclusive codes: 0 = no attention for the toy, 1 = committed compliance (child looks but does not touch the toy), 2 = situational or shaky compliance (the child touches the toy during a part of the segment), 3 = non-compliance or deviation (the child touches the toy during the whole segment), and 4 = defiance (the child protests, becomes angry or sad). Interrater reliability coefficient (ICC; based on 27 videos) was α = 0.92. For the analyses, the proportion of inhibitory control was calculated by dividing the number of segments the infant showed committed compliance (code 1) by the number of segments the child showed attention for the toy (code 1–4; Kochanska et al., 1998). For 24 children we had no data on the inhibitory control task, because mother and child did not participate in the data wave at 12 months (*n* = 17), the child was not interested in the toy (*n* = 3) or the child became upset during the task (*n* = 3), or there were problems with the video recording (*n* = 1).

#### Inhibitory Control at 30 Months

At 30 months, inhibitory control was measured using the Gift delay task (Kochanska et al., 2000). At the end of the assessment, the experimenter praised the child and put a gift box (including a present) on the table in front of the child. The child was asked to wait and not to take the present out of the box, whilst the experimenter left them alone for 3 min. Child behavior was videotaped and coded afterward using five codes: 0 = opens the box and takes the present, 1 = opens the box and takes the present, but puts it back, 2 = opens and peeks inside the box, 3 = touches the box, 4 = does not touch the box. For the analyses, code 0 and 1 were merged, because only four children scored code 1. Missing values were due to mother-child dyads not participating in the assessment at 30 months (*n* = 35), children’s inability to understand the task (*n* = 2) or becoming upset (*n* = 1).

#### Vocabulary at 12 Months

Vocabulary at 12 months was measured using the Dutch adaptation of the MacArthur-Bates Communicative Development Inventories: Words and Gestures 1 (NCDI-1 short form; Fenson et al., 2000; Zink and Lejaegere, 2003). Mothers were asked to tick the sounds and words the child comprehended (vocabulary comprehension) and the sounds and words the child used (vocabulary production). Since the number of meaningful words children can produce during infancy is limited, this study used the total number of words the child comprehended in the analyses (possible range: 0–103). Twenty-three children did not have a score on vocabulary, because mother and child did not participate in the data wave (*n* = 17) or did not fill out the backside of the questionnaire (*n* = 6).

#### Vocabulary at 30 Months

Vocabulary comprehension at 30 months was measured using the Dutch adaptation of the MacArthur-Bates Communicative Development Inventories: Words and Gestures 2a (NCDI-2a; short form; Fenson et al., 2000; Zink and Lejaegere, 2003), that has similar procedures as the NCDI-1 short form. The possible range of vocabulary comprehension reported by the mother was 0–122. Missing data on vocabulary at 30 months (*n* = 32) was due to not participating in the assessment at 30 months (*n* = 35; but five mothers completed the questionnaire at home) or because the child was judged (by the mother) not to have reached the minimum score (*n* = 2).

#### Attention at 12 Months

Sustained attention was examined at 12 months using an adapted version of the Task orientation paradigm (Goldsmith and Rothbart, 1999). While the child was sitting in a high chair, a music box was placed on the table out of reach of the infant for 2 min. The experimenter asked the mother, who sat behind the infant, not to interfere. For the analyses, the amount of time the infant looked at the music box was coded afterward from videotapes (possible range 0–120 s). ICC was α = 0.99 (based on 27 videos). Twenty-six children did not have data, because mother and child did not participate in the data wave at 12 months (*n* = 17), the task was aborted because the child became upset (*n* = 8), or the task was not filmed (*n* = 1).

#### Attention at 30 Months

Sustained attention at 30 months was examined using the same Task orientation paradigm (Goldsmith and Rothbart, 1999), as described for the 12 months assessment. ICC of coding the amount of time the infant looked at the music box was α = 0.99 at 30 months (based on 30 videos). Thirty eight children had missing data on the attention task at 30 months, because mother and child did not participate in the assessment at 30 months (*n* = 35), the task was not filmed due to technical problems (*n* = 1), or the children were unable to understand or perform the task (*n* = 2).

### Data Analyses

First, descriptive statistics and correlations between the study variables were examined using the Statistical Package for the Social Sciences (SPSS; version 25). Outliers (>3 SD from the mean) detected for physical aggression at 12 months (*n* = 1), 20 months (*n* = 4) and 30 months (*n* = 1), vocabulary at 30 months (*n* = 4), and attention at 30 months (*n* = 6) were winsorized to the values three standard deviations from the mean.

The Mplus statistical software package was used to examine trajectories of physical aggression, including physical aggression scores at 12, 20, and 30 months (Muthén and Muthén, 1998–2017). To examine the first hypothesis, a latent growth curve model was estimated to examine the variance of the intercept and linear slope of the mean trajectory of physical aggression. Significant variance indicates that the participants do not follow one specific aggression trajectory and justifies identifying different trajectories of aggressive behavior. Acceptable fit of the latent growth curve model was indicated by a non-significant Chi-square test, a Tucker-Lewis index (TLI) and a comparative fit index (CFI) higher than 0.95, a root mean-square error of approximation (RMSEA) below 0.06, and a standardized root mean-square residual (SRMR) below 0.08 (Hu and Bentler, 1999).

Next, a series of unconditional latent class growth analyses were performed to identify different trajectories of aggressive behavior between 12 and 30 months. Participants with physical aggression data on at least two data waves were included in the analyses (*n* = 172). Models with one to five classes were estimated. As recommended by Nylund et al. (2007) and Jung and Wickrama (2008), the final number of classes was determined by the Bayesian information criteria (BIC), the Vuong-Lo-Mendell-Rubin likelihood ratio test (VLMR-LRT), the bootstrap likelihood ratio test (BLRT), the entropy index, the posterior probabilities (PP) for class membership, and the proportion of participants in each class. A lower value of the BIC indicates a better fit. In addition, a significant VLMR-LRT and BLRT both indicate a better fit of the tested model compared to the more parsimonious model (Nylund et al., 2007; Jung and Wickrama, 2008). An entropy index and PP for class membership close to 1.0 indicate clear classification of the participants in the classes. It is recommended that each class has at least 1% of the participants (Frankfurt et al., 2016). A study comparing the BIC and LRTs showed that the BLRT was the best indicator to identify the correct number of classes, followed by the BIC (Nylund et al., 2007). After the number of classes was determined, the shapes of the trajectories in the final model were examined.

The latent growth curve model and latent class growth analyses were performed using full information maximum likelihood (FIML) with robust standard errors in case of missing data (Muthén and Muthén, 1998–2017). Because of unequal time intervals between assessments, time scores for slope growth factor were set at 0, 8, and 18, corresponding to the time intervals between the data waves.

After the final model of the latent class growth analyses was selected, participants were assigned to the trajectory for which the child had the highest posterior probability estimate, which indicates the likelihood a child would follow a certain trajectory. To examine to second hypothesis, a multinomial logistic regression analysis was conducted using SPSS to predict group assignment by the cognitive predictors at 12 months: inhibitory control, attention, and vocabulary. The low physical aggression trajectory was used as reference category.

To test the third hypothesis, a series of repeated measures analyses of variance were conducted to compare cognitive development between age 12 and 30 months (no 20-month assessments of relevant aspects of cognition available) between the children following different trajectories of physical aggression. The low-stable group was used as comparison group. Because inhibitory control at 12 and 30 months were measured using two different tasks, scores on inhibitory control were converted to *z*-scores prior to the repeated measures analyses.

Gender was examined as potential covariate for the multinomial logistic regression analysis and repeated measures analyses by examining the relation with the physical aggression trajectories using Chi-square test. Statistical significance was set at *p* < 0.05.

## Results

### Preliminary Analyses

Descriptive statistics and correlations between the study variables are reported in Table 1. The Supplementary Materials include boxplots of the study variables (with individual data points overlaid).

**TABLE 1:** Descriptive statistics and correlation analyses for study variables (*n* = 182).

	1	2	3	4	5	6	7	8	9
1. Inhibitory control 12 months	–								
2. Inhibitory control 30 months	0.12	–							
3. Attention 12 months	0.13	0.05	–						
4. Attention 30 months	–0.07	–0.07	0.13	–					
5. Vocabulary 12 months	0.03	0.02	–0.03	–0.01	–				
6. Vocabulary 30 months	0.04	–0.02	0.12	0.01	0.44**	–			
7. Physical aggression 12 months	−0.19*	0.01	–0.13	0.11	–0.00	–0.08	–		
8. Physical aggression 20 months	−0.18*	–0.15	−0.22**	–0.06	–0.08	–0.12	0.33**	–	
9. Physical aggression 30 months	–0.14	−0.27**	–0.05	0.18*	–0.12	−0.27**	0.27**	0.45**	–
*n*	158	144	156	144	159	150	172	177	150
*M*	0.33	1.48	58.95	98.15	42.65	104.88	2.67	2.64	2.31
SD	0.32	0.95	22.50	20.96	21.75	10.33	1.84	2.30	2.16
Min	0.00	0.00	0.00	30.00	0.00	71.00	0.00	0.00	0.00
Max	1.00	3.00	116.00	120.00	103.00	112.00	8.46	9.92	8.95

*p < 0.05, **p < 0.01.

### Main Analyses

To examine the first hypothesis, an unconditional linear growth curve model and a series of unconditional latent class growth analyses were conducted. The unconditional linear growth curve model was run to examine the variance of the intercept and linear slope of the mean trajectory of physical aggression between 12 and 30 months. The data fitted the model well, χ^2^(1) = 0.65, *p* = 0.42; TLI = 1.00; CFI = 1.00; RMSEA < 0.01, 90% CI (0.00;0.18); SRMR = 0.02. On average, the estimated means indicated that physical aggression remained relatively stable between 12 months (*M* = 2.67, SD = 2.07) and 20 months (*M* = 2.65, SD = 2.32), followed by a decrease up to 30 months (*M* = 2.34, SD = 2.15). The linear slope indicated that the decrease between age 12 and 30 months was not significant: intercept = 2.70, SE = 0.14, *p* < 0.01; linear slope = −0.02, SE = 0.01, *p* = 0.08. The variance of the intercept (σ^2^ = 1.65, SE = 0.55, *p* < 0.01) and linear slope (σ^2^ < 0.01, SE < 0.01, *p* = 0.03) were significant, indicating diversity in children’s start level and slope, which justifies examining different physical aggression trajectories.

Next, a series of unconditional latent class growth analyses was conducted to identify the optimal number of trajectories for physical aggression between age 12 and 30 months. Participants with data on at least two data waves were included (*n* = 172). Based on the different fit indices shown in Table 2, the best fitting model included four classes. The BIC decreased up to the 4-class model and increased for the 5-class model, indicating best fit for the 4-class model. The BLRT remained significant for the 2-, 3-, and 4-class model, but was not significant for the 5-class model, indicating preference for the more parsimonious 4-class model. The results of the VLMR-LRT were in the same direction: the significant VLMR-LRT for the 2-class model indicated that the 2-class model was preferred above the 1-class solution. For the 3- and 4-class models the VLMR-LRT was a trend. The non-significant VLMR-LRT for the 5-class model indicated no significant improvement of the model. Moreover, the entropy (0.81) and the mean posterior probability scores (ranging from 0.84 to 0.91) of the 4-class solution indicated that the classes were separated well.

**TABLE 2:** Solutions of the series of latent class growth analyses.

	BIC	BLRT	VLMR-LRT	Entropy	PP	% in classes
1-class model	2129.46	N/A	N/A	1.00	1.00	100.0
2-class model	2070.18	*p* < 0.01	*p* < 0.01	0.80	0.89	20.3
					0.95	79.7
3-class model	2062.42	*p* < 0.01	*p* = 0.09	0.84	0.87	20.3
					0.94	76.7
					0.93	2.9
4-class model	2045.29	*p* < 0.01	*p* = 0.05	0.81	0.84	12.2
					0.91	65.7
					0.91	3.5
					0.89	18.6
5-class model	2052.91	*p* = 0.13	*p* = 0.30	0.80	0.97	2.3
					0.90	61.6
					0.92	9.9
					0.74	5.2
					0.77	20.9

*BIC, Bayesian information criterion; BLRT, bootstrap likelihood ratio test; VLMR-LRT, Vuong-Lo-Mendell-Rubin likelihood ratio test; PP, posterior probabilities.*

**FIGURE 1:**
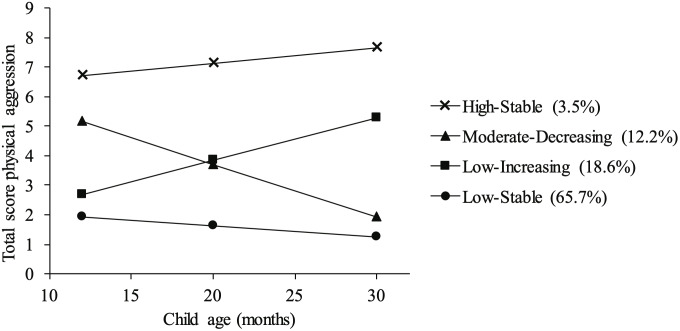
The trajectories of physical aggression of the 4-class model.

The physical aggression trajectories of the 4-class model are shown in Figure 1. The unstandardized growth parameters (intercept and linear slope) of the different trajectories are presented in Table 3. The majority of the children were assigned to the low-stable group (*n* = 113, 65.7%). This group had the lowest levels of aggressive behavior at 12 months. Although the parameters indicated that physical aggression significantly decreased in this trajectory, the absolute change was limited compared to the other trajectories. For that reason, this group was named the low-stable trajectory. The second class was labeled as the low-increasing trajectory (*n* = 32, 18.6%), which showed relatively low levels of physical aggression at 12 months that significantly increased until the children were 30 months. The third class was named the moderate-decreasing trajectory (*n* = 21, 12.2%). This group exhibited moderate levels of physical aggression at 12 months, which significantly decreased until the children were 30 months old. The final class was the high-stable trajectory (*n* = 6, 3.5%), with children showing a persistent level of high aggressive behavior between 12 and 30 months. Because the high-stable group only consisted of six children, the results of analyses including the high-stable group are not reported due to a lack of statistical power.

**TABLE 3:** Growth parameters (intercept and linear slope) of the trajectories of physical aggression.

	*n*				
	Total	Boys	Girls	Intercept (SE)	Linear slope (SE)
Low-stable	113	54	59	1.91 (0.23)**	−0.04 (0.01)**
Low-increasing	32	18	14	2.69 (0.26)**	0.14 (0.02)**
Moderate-decreasing	21	11	10	5.15 (0.71)**	−0.18 (0.04)**
High-stable	6	6	0	6.72 (0.97)**	0.05 (0.05)

*p < 0.05, **p < 0.01.

Next, gender was tested as potential covariate. Because gender was not significantly related to the aggression trajectories, χ^2^(2) = 0.77, *p* = 0.68, gender was not included in the subsequent analyses.

For the second hypothesis, a multinomial logistic regression analysis with inhibitory control, attention, and vocabulary at 12 months as predictor variables was conducted to predict assignment of the children to the different trajectories of physical aggression. The low-stable trajectory was used as the reference group in the multinomial logistic regression analysis. The descriptive statistics of cognition and the results of the multinomial logistic regression analysis are shown in Tables 4, 5, respectively. The final model of the multinomial logistic regression analysis (−2log likelihood = 223.69) fit the data better compared to the intercept only model (−2log likelihood = 242.48), χ^2^(6) = 18.79, *p* < 0.01; Nagelkerke’s *R*^2^ = 0.15; Cox and Snell’s *R*^2^ = 0.13).

**TABLE 4:** Descriptive statistics of cognition at 12 and 30 months for the low-stable, low-increasing, and moderate-decreasing trajectory groups.

	Physical aggression trajectories					
	Low-stable		Low-increasing		Moderate-decreasing	
	*M*	SD	*M*	SD	*M*	SD
Inhibitory control 12 months	0.38	0.34	0.34	0.29	0.18	0.23
Inhibitory control 30 months	1.60	0.91	1.20	1.03	1.50	0.97
Attention 12 months	63.20	21.13	55.00	24.71	50.76	24.30
Attention 30 months	96.76	22.73	103.33	13.36	95.49	23.57
Vocabulary 12 months	45.39	21.97	34.73	20.52	43.57	22.31
Vocabulary 30 months	106.58	8.64	99.62	13.57	104.76	10.91

**TABLE 5:** Multinomial logistic regression analyses examining inhibitory control, attention, and vocabulary at 12 months as predictors of the physical aggression trajectories.

	Low-increasing			Moderate-decreasing		
	*B*	SE	OR	*B*	SE	OR
Constant	0.85	0.81		0.74	0.88	
Inhibitory control	–0.26	0.72	0.77	−2.50*	1.12	0.08
Attention	–0.02	0.01	0.98	−0.02*	0.01	0.98
Vocabulary	−0.03*	0.01	0.98	–0.01	0.01	1.00

OR, odds ratio. The low-stable physical aggression trajectory was used as reference group. *p < 0.05, **p < 0.01.

Vocabulary at 12 months significantly predicted assignment to the low-increasing trajectory compared to the low-stable trajectory, *B* = −0.03, SE = 0.01, OR = 0.98, *p* = 0.03. The odds ratio’s indicated that lower vocabulary led to a higher chance of being in the low-increasing compared to the low-stable trajectory. Attention and inhibitory control at 12 months did not predict group membership in the low-increasing group.

Classification in the moderate-decreasing versus low-stable trajectory was significantly predicted by inhibitory control, *B* = −2.50, SE = 1.12, OR = 0.08, *p* = 0.03, and attention at 12 months, *B* = −0.02, SE = 0.01, OR = 0.98, *p* = 0.049. Children with lower inhibitory control or attention scores were more likely to show the moderate-decreasing trajectory versus the low-stable trajectory of physical aggression. Vocabulary at 12 months did not predict membership of the moderate-decreasing group.

**FIGURE 2:**
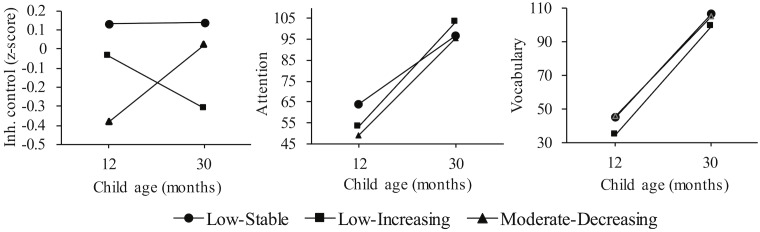
Development of cognition for the low-stable, low-increasing, and moderate-decreasing physical aggression trajectories. Inh. Control, inhibitory control.

Regarding the third hypothesis, the developmental patterns of inhibitory control, attention, and vocabulary between age 12 and 30 months were examined for the three physical aggression trajectories using a series of repeated measures analyses. Because inhibitory control was measured using different scales at 12 and 30 months, scores were converted to *z*-scores. The low-stable group was used as comparison group. Figure 2 shows the developmental patterns of cognitive functioning between 12 and 30 months for the physical aggression trajectories.

Repeated measures analysis examining inhibitory control for the low-increasing and low-stable groups indicated a trend for the main effect of trajectory group, indicating that the low-increasing group had lower scores on inhibitory control compared to the low-stable group from age 12 up to 30 months, *F*(1,110) = 3.40, *p* = 0.07, $\eta_{p}^{2}=0.03$. No interaction effect between age and trajectory group was found, indicating the marginal difference between low-stable and low-increasing groups regarding inhibitory control remained stable. With respect to attention, no main effect of trajectory group was found. A significant interaction between age and trajectory group indicated a larger increase in attention for the low-increasing group compared to the low-stable group, *F*(1,107) = 7.34, *p* < 0.01, $\eta_{p}^{2}=0.06$. Figure 2 indicates that the low-stable scored higher in attention at 12 months, which was not found at 30 months. For vocabulary, repeated measures analysis indicated a significant main effect for trajectory group, *F*(1,114) = 9.15, *p* < 0.01, $\eta_{p}^{2}=0.07$, showing lower scores on vocabulary for the low-increasing compared to the low-stable group at ages 12 and 30 months. No significant interaction between age and trajectory group was found.

When comparing the moderate-decreasing group with the low-stable group, repeated measures analyses indicated no significant main effects of trajectory group or interaction effects including inhibitory control or vocabulary. For attention, a trend was observed for both the main effect of trajectory group, *F*(1,97) = 3.27, *p* = 0.07, $\eta_{p}^{2}=0.03$, and the age-group interaction, *F*(1,97) = 3.46, *p* = 0.07, $\eta_{p}^{2}=0.03$: the moderate-decreasing group had lower scores in attention compared to the low-stable group, but also showed a greater improvement in attention from age 12 to 30 months. Figure 2 shows that the difference in attention found at 12 months was not present anymore at 30 months.

## Discussion

To our knowledge, this is the first study investigating the role of early cognition in the development of physical aggression from infancy to toddlerhood. Four distinctive trajectories of physical aggression were identified: whereas the majority of children followed a low-stable trajectory, other children showed a low-increasing, moderate-decreasing, or high-stable pattern of physical aggression between age 12 and 30 months. Lower inhibitory control, vocabulary and attention at 12 months selectively predicted the likelihood of following the moderate-decreasing or low-increasing physical aggression trajectories compared to the low-stable pattern. The development of inhibitory control, vocabulary, and attention between age 12 and 30 months was also (marginally) related to the low-increasing or moderate-decreasing physical aggression trajectories compared to the low-stable trajectory.

### Physical Aggression Trajectories

Based on research focusing on physical aggression trajectories starting at 1.5 years old, three trajectories were expected: low-stable, moderate-increasing, and high-increasing/high-stable trajectories of physical aggression (Huijbregts et al., 2008; Wildeboer et al., 2015; Mazza et al., 2017). In addition to these trajectories, we found a fourth pattern of physical aggressive behavior starting at 12 months: 12.2% of the children started with moderate levels of physical aggression at 12 months, followed by a decrease in their levels of physical aggression to 30 months. Lorber et al. (2018) also showed that not all individual physical aggressive behaviors have the expected increase in frequency during early development. In their sample, aggressive behaviors such as biting, pulling, and pinching became less common between 8 and 24 months. The current results are in agreement with these findings: there appears to be a group of children in whom certain, rather overt aggressive behaviors, such as biting, pinching, and pulling, decrease from a very young age onward. It is unclear what underlies this pattern: possibly these children show these specific aggressive acts more as a form of exploratory behavior, and not because they are driven by fear or anger or by achieving goals. As far as possible, examination of the emotional state, goals, and intentions underlying aggressive behavior in young children might give more insight in the explanatory factors of the developmental pattern. It is also possible that many parents act more boldly or thoroughly when confronted with these specific aggressive behaviors, thereby erasing them more swiftly than other less overt forms of (physical) aggression. A third possibility is that parents, who filled out the questionnaire on physical aggression, under-reported such overt forms of physical aggression when their children get older as they believe such behaviors no longer meet societal norms. Apart from these potential explanations for the existence of a moderate-decreasing trajectory of physical aggression, the current results also suggest a role for cognitive maturation in this pattern of behavioral change (Adolph and Berger, 2015). The decrease in aggressive behavior could also (in part) be explained by development of other aspects of self-regulation (apart from cognitive maturation), such as emotional regulation. Emotional regulation radically develops from complete dependence on caregivers for regulation during the first months of life, via co-regulation during the second half of the first year to more independent and active self-regulation in toddlerhood (Calkins and Keane, 2009). Children learn to use strategies such as reaching, redirecting, self-soothing, and private speech to regulate emotional arousal. The development of cognition and emotion regulation are the results of brain development during the first years of life, especially in the prefrontal regions and limbic systems (Callaghan and Tottenham, 2016).

### Relation Between Cognition and Physical Aggression Trajectories

The low-increasing trajectory group initially showed low levels of physical aggression at 12 months, followed by an increase in physical aggression up to 30 months. In line with previous research showing a relation between less developed cognition and elevated levels of aggressive behavior during preschool (e.g., Estrem, 2005; Spann and Gagne, 2016), the results of the current study suggest that cognition during infancy and toddlerhood can be associated with increasing patterns of physical aggression. Children with poorer vocabulary abilities at 12 months and less advantageous developmental patterns of vocabulary and inhibitory control between age 12 and 30 months were more likely to follow the low-increasing trajectory compared to the low-stable trajectory.

The moderate-decreasing group showed moderate levels of physical aggressive behavior at 12 months, decreasing to typical levels of physical aggression up to 30 months. The likelihood to follow the moderate-decreasing versus the low-stable physical aggression trajectory was predicted by lower levels of inhibitory control and attention at 12 months. This decline in physical aggression was also marginally related to maturation of attention from age 12 months up to 30 months. As shown in Figure 2, the absolute differences in attention at 12 months between the moderate-decreasing and low-stable group became smaller at 30 months. These results suggest that while attention improved to the levels of the low-stable group from age 12 up to 30 months, physical aggression normalized in this period to a low level for this group of children.

In line with previous research examining older children (Tremblay et al., 2004; Campbell et al., 2006), the results of this study indicated a high-stable group showing persistent levels of high physical aggression during infancy and toddlerhood. The high-stable group consisted only of six children (3.5%). As this could raise concerns regarding the limited power when examining (cognitive) correlates of high-stable physical aggression, no such statistical analyses were performed with the high-stable-group. However, the four-class model for physical aggression still provided the best fit for the data. Also the proportion of children in the high-stable trajectory was similar to that observed in other studies focusing on toddlerhood and early preschool age (Campbell et al., 2006; Wildeboer et al., 2015; Olson et al., 2017). For example, Olson et al. (2017) found high chronic trajectory from 3 to 10 years with only 9 out of 218 children (3.8%).

### Strengths and Limitations

A clear strength of this study is its longitudinal design, examining both physical aggression and cognition over time. Second, this is, to our knowledge, the first study examining distinct trajectories of physical aggression starting at the age of 12 months. Next, performance tasks (except for vocabulary) were used to examine the role of infant cognition in the development of aggression.

In addition to these strengths, several limitations should be mentioned. The low power, particularly regarding the high-stable aggression trajectory, was already noted but seems to be the most important limitation. The marginal effects that were found should therefore be interpreted with caution. Another limitation may be that, although previous studies provided evidence for non-linear development of physical aggression (Nærde et al., 2014), this study only examined linear trajectories due to limited time points. Future studies can include more time points and use non-linear (e.g., quadratic or cubic) analyses to get a more comprehensive account of the different physical aggression trajectories during infancy and toddlerhood. Although previous research has shown that gender is related to the development of physical aggression and cognition during early childhood (Hay, 2017), this study did not examine physical aggression trajectories for boys and girls separately, which would be recommended for future research using larger sample sizes. In addition, all constructs in this study were examined using a single instrument. Future studies may consider the use of multiple instruments to measure each construct, thereby taking into account feasibility (e.g., with respect to concentration levels and time investment) for such a young group of participants. Mothers were used as informant to assess physical aggression, as well as vocabulary. This approach may have inflated the associations between vocabulary and physical aggression due to common method variance (Doty and Glick, 1998; Neyer, 2006). Future studies may consider using an actual test paradigm for the assessment of language as well, or use an observational method to assess aggression. Use of multiple informants might also help prevent common method variance. As for children of this age their parents are probably the most reliable informants, fathers may be most suitable as further observants (Dave et al., 2008). The measures used to examine inhibitory control involved “hot” aspects of cognition, as they incorporated affective and motivational elements. In addition, the paradigm used to measure attention may have elicited frustration in the children, as the task was lengthy without any novel elements and children were not able to play with the music box. Effects of physiological and emotional arousal cannot be ruled out in this study, although *post hoc* analyses for inhibitory control indicated comparable associations (between task performance and aggression) when children who became visibly upset were excluded from statistical analyses. In addition, only pregnant women between 17 and 25 years were eligible to participate in this study, which might imply that the results of this study are not easily applicable to children of older women. It may also rightfully be argued that there are many different risk factors influencing young children’s chances to develop physically aggressive behavior. Whereas this can obviously not be denied we chose to focus on child factors that may be more malleable (e.g., in interventions) than the majority of established risk factors.

### Implications

Previous research has indicated that high and persistent levels of physical aggression are related to adverse developmental outcomes, such as internalizing and externalizing behavior problems, criminal behavior and lower academic achievement (Mesman et al., 2001; Broidy et al., 2003; Masten et al., 2005). This study indicated that the developmental trajectories of increasing and high stable physical aggression may (already) originate during infancy. The majority of recommendations state that interventions should be deployed as early as possible (Doyle et al., 2009). The very early start of disadvantageous physical aggression trajectories observed in this study supports this stance. The results also show a potentially important role of early cognition in the development of physical aggression. This implies that interventions aimed at reducing the chances of a poor early start regarding behavioral development could involve targeting early cognitive development.

## Data Availability Statement

The datasets generated for this study are available on request to the corresponding author.

## Ethics Statement

The studies involving human participants were reviewed and approved by the ethics committee of the Department of Education and Child Studies at the Faculty of Social and Behavioral Sciences, Leiden University, and the Medical Research Ethics Committee at Leiden University Medical Centre, Leiden University. Written informed consent to participate in this study was provided by the participants’ legal guardian/next of kin.

## Author Contributions

SH, KH, SG, and HS designed the project. DA conducted the data collection. DA and SH performed and interpreted the statistical analyses and wrote the first draft of the manuscript. All authors discussed the results, contributed to the manuscript revision, and read and approved the submitted version.

## Conflict of Interest

The authors declare that the research was conducted in the absence of any commercial or financial relationships that could be construed as a potential conflict of interest.
