# Supplementary material for: The Role of Inhibitory Control, Attention and Vocabulary in Physical Aggression Trajectories From Infancy to Toddlerhood
Source: Front Psychol. 2020 May 26;11:1079. doi: 10.3389/fpsyg.2020.01079 (PMC7264375; doi:10.3389/fpsyg.2020.01079)
Supplement: Supplementary file 1 [file Image_1.PDF]

Supplementary Material

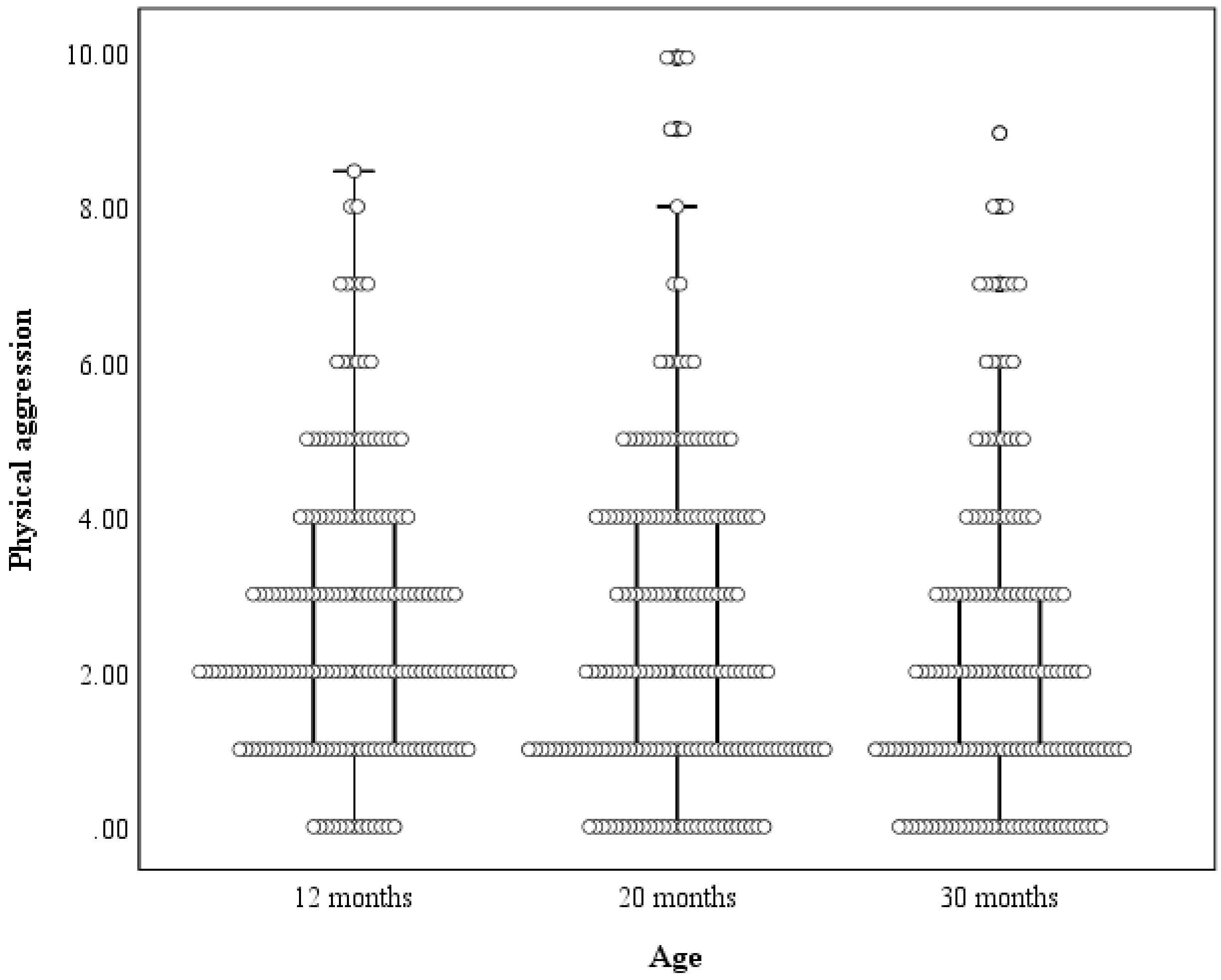

Supplementary Figure 1. Boxplots (with individual data points overlaid) of physical aggression at 12, 20 and 30 months.

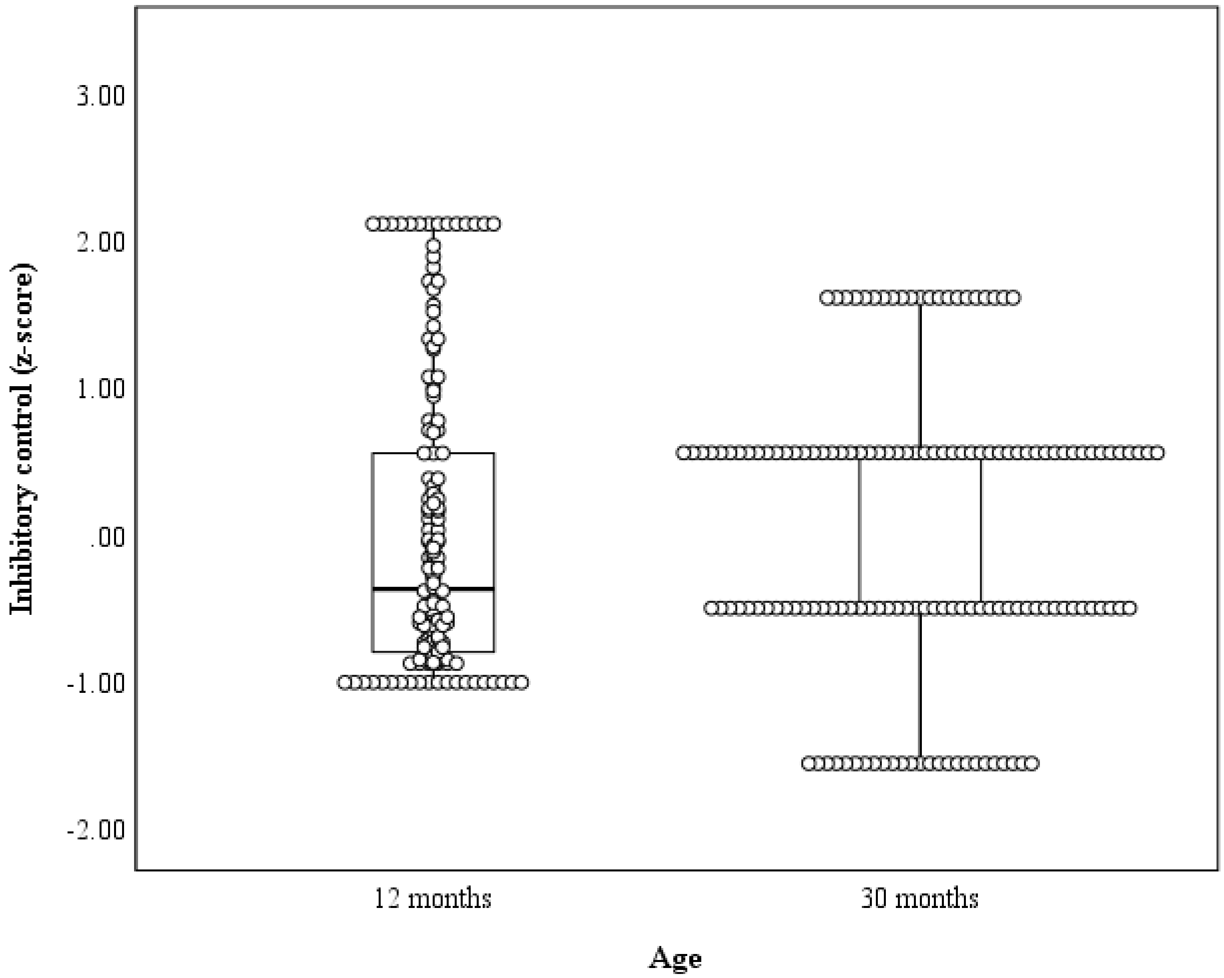

Supplementary Figure 2. Boxplots (with individual data points overlaid) of inhibitory control at 12 and 30 months.

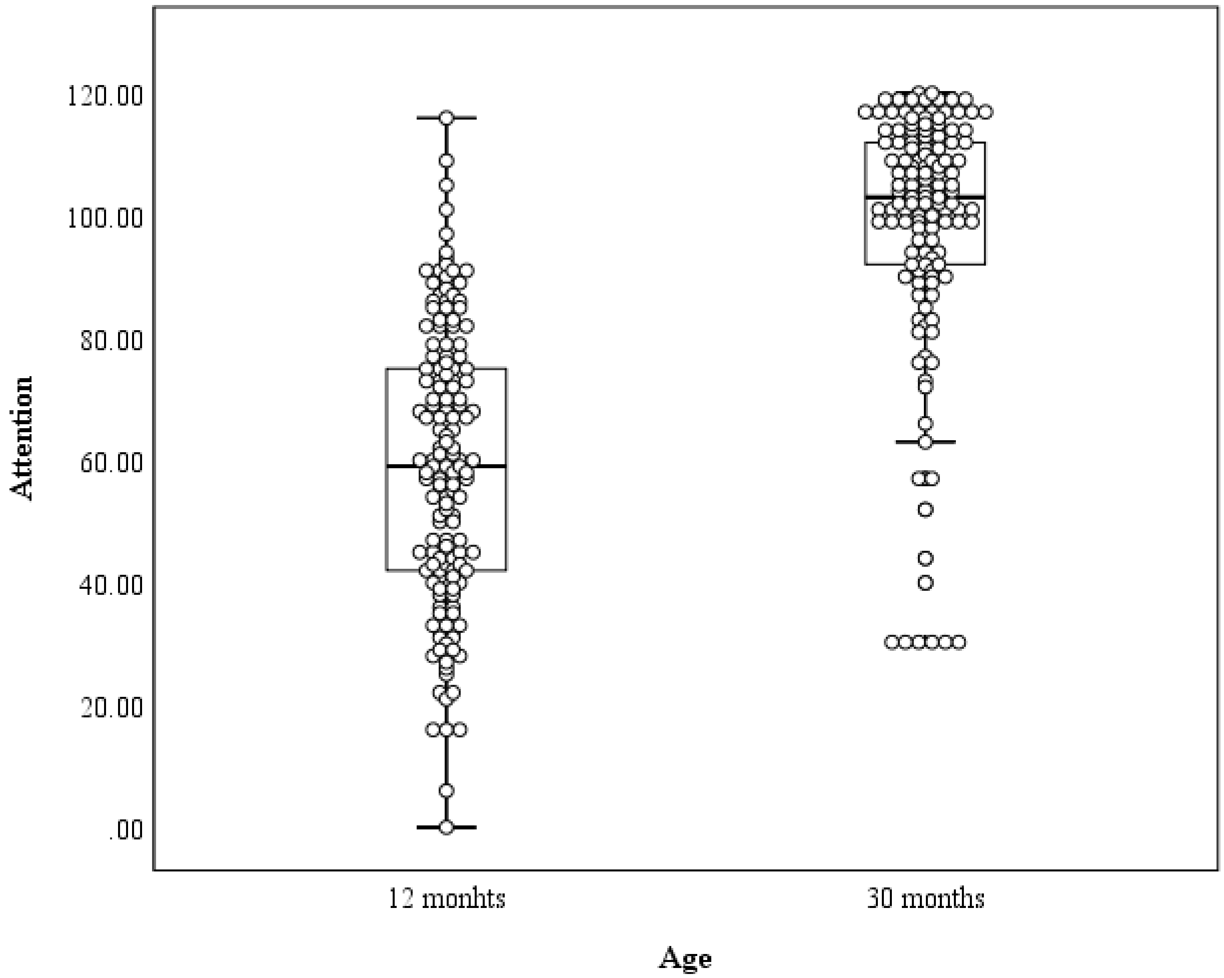

Supplementary Figure 3. Boxplots (with individual data points overlaid) of attention at 12 and 30 months.

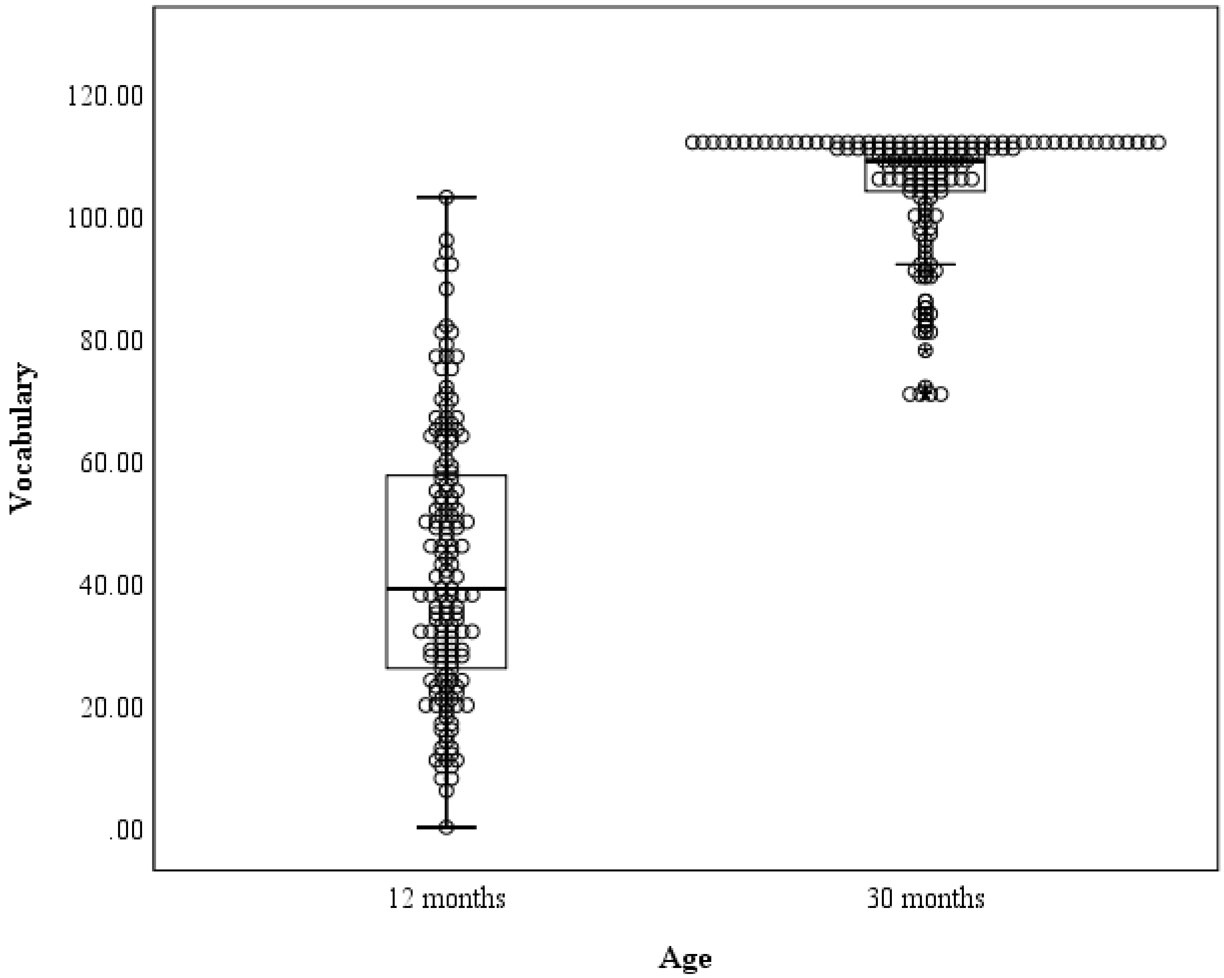

Supplementary Figure 4. Boxplots (with individual data points overlaid) of vocabulary at 12 and 30 months.
